# Researchers’ perspectives on the integration of molecular and genomic data into malaria elimination programmes in Africa: a qualitative study

**DOI:** 10.1186/s12936-024-05205-5

**Published:** 2024-12-18

**Authors:** Paulina Tindana, Daniel Enos Sekwo, Leonard Baatiema, Abdoulaye Djimde, the Pathogen Genomics Diversity Network, Africa (PDNA)

**Affiliations:** 1 Department of Health Policy, Planning and Management, School of Public Health, College of Health Sciences, University of Ghana, Accra, Ghana; 2 Malaria Research and Training Center, Faculty of Pharmacy, University of Sciences, Techniques and Technologies of Bamako, Bamako, Mali; 3 Pathogen Genomics Diversity Network Africa (PDNA), Bamako, Mali

**Keywords:** Genomics, Malaria control, Molecular surveillance, Malaria elimination, Policy decision making, Evidence translation

## Abstract

**Background:**

Malaria remains a significant public health concern, despite global efforts to combat the disease with highest burden in Africa. Reports of emerging artemisinin partial- resistance in East Africa emphasize the importance of molecular data to guide policy decisions. Hence the need for researchers to collaborate with National control programmes to conduct genomics surveillance of malaria to inform malaria control and elimination policies. This study explored genomic researchers’ views on engaging with national control programmes to aid malaria elimination efforts in Africa.

**Methods:**

This research employed an exploratory qualitative approach to investigate the views and experiences of malaria genomics researchers across 16 member countries of the Pathogen Genomic Diversity Network Africa (PDNA). In-depth interviews were conducted with each PDNA Principal Investigator, which were recorded, and transcribed verbatim. Subsequently, the data were analysed thematically with NVivo 12 qualitative data analysis software.

**Results:**

The study revealed that majority of malaria genomics researchers focused on understanding the genetic composition and adaptation of the malaria parasite, its vector, and human host. Their investigations delved into areas such as drug and insecticide resistance, parasite evolution, host interactions, human host susceptibility to malaria, diversity of vaccine candidates, and molecular surveillance of malaria. Challenges included limited funding, lack of interest and capacity among National Malaria Control Programmes (NMCP) to use research evidence effectively, and difficulties in communicating data implications to policymakers due to the absence of WHO-certified use cases. Despite these obstacles, researchers expressed a keen interest in forming partnerships with NMCPs to integrate genetic data into malaria control efforts in Africa. They also stressed the importance of enhancing researchers' ability to communicate findings to policymakers and local communities through policy briefs and innovative communication strategies.

**Conclusion:**

The study underscores the need to strengthen partnerships between genomic researchers and NMCPs to support malaria elimination in Africa. Furthermore, researchers should create practical frameworks for easy integration into WHO reporting formats to facilitate the use of molecular and genomic data in malaria control programme decision-making.

## Background

The past decades have witnessed an unprecedented array of renewed political and financial commitment, including interventions and initiatives to reduce the burden of malaria globally [1]. Some of these interventions include insecticide-treated nets, indoor residual spraying (IRS), intermittent preventive treatment in pregnancy (IPT_P_), and seasonal malaria chemoprevention (SMC) [1–3]. This notwithstanding, malaria remains a leading cause of morbidity and mortality globally with about 249 million cases reported in 2022 in 84 malaria endemic countries [2]. Sadly, sub-Saharan Africa (SSA) continues to lead in the global burden of malaria.

Several factors have been attributed to the exceedingly high burden of malaria in SSA despite investments in programmes to prevent and control the disease. Notable among these factors is the inherent ability of *Plasmodium falciparum*, the parasite responsible for most malaria in SSA to develop resistance to interventions leading to genetic threats, such as drugs and diagnostic resistance, poor uptake of contextual malaria molecular surveillance (MMS) evidence for control decision-making and limited funding for sustained high impact interventions, such as IRS [4, 5]. The weak and uninvested nature of the health systems in Low and Middle Income Countries, recurrent evolution of insecticide and drug resistance are also key drivers of the high burden of malaria in Africa [6]. Evidence indicates that the effectiveness of insecticide-treated nets (ITNs) has not reached its full potential, largely due to the discrepancy between high ITN distribution rates among vulnerable populations like pregnant women and children and actual usage rates [7, 8]. Conversely, targeted interventions such as IPT_P_ and SMC have demonstrated significant impact. However, the high costs associated with these interventions pose a threat to their widespread implementation, compounded by policymakers’ reluctance to incorporate new interventions into national health policies without robust scientific evidence regarding safety, efficacy, and cost-effectiveness [9, 10]. Against this backdrop, most countries in SSA continue to face low uptake and scale up of high-impact interventions to achieve high coverage and interrupt malaria transmission. To address the genetics threats, malaria experts have suggested the need to promote the integration of genomics information within the decision-making frameworks of National Malaria Control Programmes (NMCPs) to increase the efficacy of these interventions and stem the tide of malaria [11]. There is also a call to strengthen local NMCP capacities to understand and utilize genomics data in policy and programmatic decision-making. Additionally, there is an emphasis on investing in initiatives aimed at developing standardized MMS use cases for effective integration into policies for malaria elimination in sub-Saharan Africa [12].

Despite these calls and recommendations, the continent continues to record an exceedingly high burden of malaria thus raising two critical questions about the translation of malaria genomics evidence into contextually appropriate and relevant policies. First, *what are the experiences of malaria genomic researchers working with NMCPs and other malaria policymakers on translating genomics data for policy decision-making* and second, *what is the level of interactions, experiences and challenges associated with translating malaria genomics data for policy decision-making*? To address these questions, this study aimed to (a) explore the experiences of malaria genomic researchers working with NMCPs and other malaria policymakers on translating genomics data for policy decision-making and (b) understand the level of interactions and the experiences of challenges associated with translating malaria genomics data for policy decision making. Addressing these questions is critical to unpacking the nuances associated with the speedy translation and uptake of genomic data in the malaria policy-making ecosystem towards reversing the high malaria burden in SSA.

## Methods

### Study design

The study employed an exploratory qualitative study design with semi-structured in-depth interviews. This approach allowed the study team to explore the level of interaction between malaria genomics researchers and NMCPs, appreciate the challenges of their interaction and document researchers’ perspectives on key recommendations for effective collaboration to promote the use of genomics data for malaria control and elimination in Africa.

### Study population

The study targeted PDNA member countries or local leads across each of the 16 countries. PDNA is an African-led Pan-African network of scientists from 16 countries working in 19 institutions and specializing in malaria molecular epidemiology (www.pathogens-dna.org). Each PDNA investigator is a leader in his/her country and most contribute to shaping national malaria policies. Others also serve on the international scene with organizations and agencies, such as the WHO-AFRO and Africa-CDC. One prominent programme of the PDNA is the Developing Excellence in Leadership and Genetics training for Malaria Elimination in sub-Saharan Africa (DELGEME, www.delgeme.org). DELGEME is a Pan-African training programme which aimed at training young African scientists in malaria genetics and bioinformatics. As part of DELGEME, PDNA engaged in a training programme specifically designed to increase the capacity of African NMCPs in genetics and molecular biology. The current study therefore interviewed these leaders to gather information on their experiences interacting with NMCPs and suggestions to improve the uptake and use of genomics data by these NMCPs for malaria control and elimination.

### Recruitment and sampling

A purposive sampling technique was employed to select all PDNA Principal Investigators (PIs) based on two main criteria: (1) as country leads of the PDNA projects and (2) close collaboration with NMCPs in their respective countries towards implementing MMS in malaria control. The study team conducted an initial online engagement as part of the activities to launch the Genomic Epidemiology for Malaria Elimination (GEME) project. Potential participants were identified following this online engagement and contacted individually for their consent and participation in the individual interviews. Principal Investigators (PIs) in countries with more than one PDNA member engaging with the NMCP were recruited based availability, while countries with single PDNA PIs were automatically enrolled in the study.

### Study tools: interview guide

A semi-structured interview guide was developed to facilitate the data collection process. Its content was informed by a review of relevant literature on malaria genomics and expertise of various PDNA PIs across the 16 member countries. The guide included sections with different questions and probes to explore the experiences of malaria genomics researchers in conducting genomics research in Africa, It also assessed the level of interaction between these researchers conducting cutting-edge Malaria Molecular Surveillance in Africa and their respective NMCPs. Other sections examined the challenges, communication strategies, the role of policy briefs in sharing information with policy makers, as well as recommendations of best practices to improve the interaction between researchers and malaria policy makers. The focus was on effectively translating and using genomics data for malaria policy decision-making across the countries.

### Data collection

Seventeen online and in-person in-depth interviews were conducted with PIs. The interviews were conducted by two members of the project team (PT and DES). With consent from study participants, all interviews were audio-recorded using a digital recorder. All interviews were conducted in English Language, and each lasted for about 45 min on average.

### Data analysis and presentation

An iterative thematic analysis was conducted in this study. All interviews were audio-recorded and transcribed verbatim. The transcripts were reviewed multiple times to identify themes, patterns, and categories. A codebook was developed in the qualitative research software Nvivo12 [13] for qualitative analysis using a combination of categories from the data in line with the study objectives. Subsequently, all transcripts were imported into the software and were coded under the pre-identified themes but also included additional codes/themes emerging from the data. These themes later formed headings for ascribing meaning and interpretation of the data. For example, the thematic analysis sought to generate evidence around the nature and focus of malaria genomics research projects, the research dissemination/translation plans of the researchers, the regularity of dissemination events, communication strategies used in communicating research findings and the challenges encountered in disseminating research findings. All results were then presented as narratives, supported with relevant and exemplar quotes from the transcripts.

## Results

### Background characteristics of study participants

Table 1 presents the socio-demographic characteristics of participants. Of the 17 participants, 70.6% (n = 12) were males. The mean age of participants was 48 years (**± **5.1 SD). All participants had received terminal degrees, and 35.3% (n = 6) had attained the rank of Professor, whilst 65% (n = 11) were either Senior Research Fellows, Principal Research Scientist or Senior Lecturers at the time of interview. Some held high positions such as Vice Chancellor, Director within their institutions. The average length of service as researchers was 12 years which is reflected in their experience engaging with policy makers (65%). In terms of direct interaction with their respective NMCPs, 88.2% (n = 15) reported they had worked directly with NMCPs while 53% (n = 9) affirmed they had experience developing policy briefs indicating a moderate level of proficiency in translating research findings into policy briefs.

**Table 1:** Socio-demographic characteristics of study participants

Characteristics	Frequency (percent)
	(N=17)
Sex	
Female	5 (29.4)
Male	12 (70.6)
Age (mean ±SD)	48.6 ±5.1
Marital status	
Divorced	1 (5.9)
Married	15 (88.2)
Single	1 (5.9)
Religion	
Christian	14 (82.4)
Muslim	1 (5.9)
None	2 (11.8)
Academic credentials	
Ass. Prof	1 (5.9)
PhD	11 (64.7)
Prof	5 (29.4)
Role in institution	
Clinical researcher	1 (5.9)
Director general of health	1 (5.9)
Lecturer	1 (5.9)
President of PDNA	1 (5.9)
Principal Scientist	1 (5.9)
Research fellow	3 (17.6)
Snr Lecturer	1 (5.9)
Snr. Research Fellow	7 (41.2)
Vice Chancellor	1 (5.9)
No of years (mean ±SD)	11.7±5.0
Ever worked directly with NMCP	
No	2 (11.8)
Yes	15 (88.2)
Experience engaging with policy makers	
Limited	3 (17.6)
No	3 (17.6)
Yes	11 (64.7)
Experience in developing policy briefs	
No	2 (11.8)
Very little	6 (35.3)
Yes	9 (52.9)

### Summary of study findings

As shown in Table 2, overall, four themes and thirteen sub-themes were generated from the data.

**Table 2:** Analysis themes and sub-themes derived from the data

Theme 1: Experience in malaria genomics research	
Sub-theme 1	Focus of genomics research studies
Sub-theme 2	Lead roles in *genomics research*
Sub-theme 3	Key findings of studies
Sub-theme 4	Policy implications of findings
Theme 2: Communicating research findings	
Sub-theme 1	Experience/frequency engaging policy makers
Sub-theme 2	Communication strategies
Sub-theme 3	Receptivity of research evidence
Theme 3: Key Challenges	
Sub-theme 1	Limited funding for research and translation
Sub-theme 2	Limited capacity
Sub-theme 3	Limited understanding of pathogen genomics by policy makers
Sub-theme 4	Translating pathogen genomics to lay audience
Sub-theme 5	Lack of interest in genomics by policy-makers
Theme 4: Recommendations to improve research uptake and dissemination	
Sub-theme 1	Education and involvement of NMCPs in research processes
Sub-theme 2	Capacity building
Sub-theme 3	Developing good working relationships
Sub-theme 4	Emulating best practices

### Experiences in malaria genomics research

The study explored the experiences of Malaria Genomic Researchers (MGRs) regarding the research projects they are engaged in within their individual countries. MGRs conveyed varying levels of experience and involvement in various aspects of malaria research. The detailed accounts provided insights into the specific focus of their research studies, the roles undertaken by the researchers, significant findings derived from their research endeavours, and the policy implications associated with these findings. This information was comprehensively explored under relevant sub-themes as follows:

### Focus of genomics research studies

MGRs shared that their research studies focus on different aspects of the three malaria genomes that are intertwined in the malaria transmission cycle. Depending on the research questions or problem under investigation, their studies could focus either on aspects of the genetics of the parasite, the human host, the vector mosquito, or their interaction. In particular, their research studies focused on understanding anti-malarial drug resistance patterns, identifying new resistance markers, testing the efficacy of front-line anti-malarial, including vaccines and other interventions, and conducting malaria molecular surveillance in collaboration with NMCPs. They highlighted other factors that shape the focus of the research they conduct including source of funding, the interest of the malaria control programme in their countries, as well as their training and areas of expertise.

“*Ok, within the last decade that we have been involved in these research activities, we have performed the genetic diversity of plasmodium parasites within the country and then we also sequenced samples through the PDNA project, we also participated in the global genome sequencing of P-falciparum samples to look for resistance markers, common markers of resistance to artemisinin*.” IDI-GR-12

“……. *Lots of them. Lots of them, suffice to say that we have done lots of projects on humans, parasites, and vectors in the past one or two decades*.” IDI-GR-03

### Lead roles in genomics research

The study participants described their evolving roles over the years within the malaria genomics epidemiology ecosystem. They described a progression of roles and responsibilities, starting with roles such as students and data collectors, advancing to research assistantships, and eventually attaining leadership roles such as principal investigators and co-investigators. This trajectory involved continuous capacity building and experiential learning, empowering them to contribute valuable research insights to inform policy. Participants emphasized the significance of their roles as researchers involved in cutting-edge collaborative malaria genomics research in Africa, underscoring the pivotal role they play in malaria control and elimination efforts on the continent.

“*So, I am the principal investigator for the Ghana component. Even though it is a collaboration between the USA and other African countries, for Ghana I am the principal investigator.”* IDI-GR-01

*“Well, my role was for data collection. The first part of the project was when I did my PhD and so I was for data collection, and thereafter I am usually the lead author in drug resistance-related papers in my institution now. And I lead the entire project as well.”* IDI-GR-08

### Key findings from genomics studies

The participants shared noteworthy findings arising from their research endeavors. These studies either contributed novel insights or supported and validated existing research evidence. Notably, in certain countries, participants’ investigations revealed markers indicating artemisinin partial resistance in specific regions. Additional reported findings encompassed alterations in the feeding habits of the vector, observed patterns in the genetics of the parasite, instances of drug ineffectiveness, and mutations identified in the vector. These findings collectively contribute to an enhanced understanding of malaria transmission dynamics and are instrumental in informing strategies for control and elimination.

“*Ok, so in brief what we have found so far is that we have a very diverse parasite population circulating in Ghana. About 7% of the vaccine’s strain is what was found in a place like Navrongo compared to about 60% found in Cape Coast. So, in general, this is the finding*.” IDI-GR-01

*“Ok, no suspected artemisinin resistant parasites have been found in the country, but we found some other markers which are a bit close to the markers associated with artemisinin resistance and isolates from west and central Africa are completely different from isolates from east Africa for example. These are the main results.* IDI-GR-03

### Policy implications of findings from genomics research

The participants expressed their perspectives on the implications of their study results for formulating malaria control strategies. They suggested that their findings could serve as crucial evidence for decision-making processes, fostering a deeper understanding of the malaria landscape in their various countries. Effective communication of this information to policymakers was deemed essential, positioning it as a valuable tool for preparedness and actionable measures. Additionally, they indicated that their results could play a role in shaping policy formulation or enhancing existing malaria policies, thereby contributing to more informed and effective strategies for tackling malaria in the sub-region.

“*If we had options of different vaccines depending on the composition or which antigen parasites were used for these different vaccines, we could look at our results and decide that since this vaccine antigen is circulating more in this region, it would work better in this region so let’s apply this vaccine for this region and this other one would be more suited for another region. If also a new vaccine is coming and they look at our data, it would give them a fair idea of their chances of success.*” IDI-GR-01

“*we share these results also in terms of helping the NMCP to update its guidelines in terms of malaria treatment so it is very important.”* IDI-GR-05

### Communicating genomic research findings

This theme explored the researchers’ experiences in communicating and translating their research findings. It covered various aspects such as the structure for malaria policy-making, the firsthand experiences of engaging with policymakers, strategies employed for communication and engagement with stakeholders, and the frequency of such engagements. Detailed discussions under these sub-themes provide valuable insights into the experiences of participants with communicating genomics findings to policy makers and the challenges therein, and strategies they employed for effectively communicating and translating malaria genomics research findings for informed policy decision-making.

### Experience engaging policy makers and frequency of engagement

Overall, participants had varying levels of experience in engaging with policymakers. The study revealed that in many countries, there was no formal structure for collaboration between MGRs and NMCPs. Consequently, MGRs had to proactively initiate and sustain working relationships with NMCPs. Some participants mentioned a communication hierarchy in their countries, where information flowed from researchers to local NMCPs and then to the Minister of Health or Director for Malaria Control. However, even in these cases, the process of feedback and uptake of research findings was reported to be slow. Most participants emphasized the significance of the relationships they established with malaria control programmes, noting that without such connections, they might submit research reports to the NMCP or Ministry of Health but receive minimal feedback beyond acknowledging receipt. Overall, participants unanimously agreed that there is substantial room for improvement in their engagement and working relationships with NMCPs, as the current experiences were deemed less than satisfactory.

*“it has been very limited I don’t really know what happens at the department of health for example, I recently did one investigation on RDT and I just wrote a report saying I did these RDT and this is their performance and these are my recommendations and then I just presented that to the National Department of Health and they thanked me very much and I walked out; that is what happens and after that I am not really sure what the next steps are. (laughs)”* IDI- GR-09

“*What I have learnt is that to incorporate genomics into malaria control or malaria elimination, you need to work together with the policy makers. You need to educate them; you need to give them a deep understanding and as I said it is an evolving field but even saying that I don’t fully understand the field is information for them. So, let’s work on it together. I think education, collaboration and working as a team. Yeah”* IDI-GR-14

The frequency of engagement and the dissemination of findings to malaria policymakers and other stakeholders Varied among participants and depended mainly on the local context. Participants who had established strong connections with their NMCPs often found it challenging to monitor the frequency of their engagements due to the ongoing nature of their collaborations. In contrast, other participants reported a more structured process where they engaged with NMCPs either annually or quarterly. However, in general, participants had minimal engagement with their communities, despite the potential benefits of disseminating research findings to community level stakeholders. Thus, underscoring an area where increased attention and involvement is required and could contribute to a more holistic and inclusive approach to research communication.

*“Yeah, quite often, actually, quite often we communicate our findings to the NMCPs very often because we have sort of regular, regular meetings with the NMCPs. Yes, we have regular meetings with NMCPs, and these are all opportunities to show our data.”* IDI-GR-08

*“Normally it is once a year but when there is something specific, we can do it at the time we find something specific.”* IDI-GR-13

### Scientific communication strategies

The participants, drawing from their diverse experiences and varying engagement experiences with malaria policymakers, highlighted the common use of conventional scientific communication strategies to convey their research findings to a wider audience. These strategies included the dissemination of research results/findings through project reports, presentations in local and international scientific conferences. The participants emphasized that these communication methods and packaging of the information must be tailored to specific audiences and target stakeholder groups. For instance, high level research findings would usually be shared within the scientific community through publications and presentations at scientific conferences. Additionally, in order to a much broader audience with diverse backgrounds, the participants utilized media platforms such as radio, television, and social media (e.g. Twitter) to disseminate the cardinal points in their research findings. The use of mass media was particularly valued by researchers for its ability to reach a broader audience.

*“But also sharing our findings on twitter is really, really one medium that reaches people so quickly and there is a wider following than we would have had during our annual research conference and so it meets a much tenfold outreach.”* IDI-GR-04

*“Actually, we have several conferences and meetings every year, and through these platforms, I usually present the findings of my research through these symposia or conferences where the NMCP and other stakeholders usually attend the meeting. The truth is that for presentation, or poster presentation, we have been sharing with all the stakeholders as far as malaria research is concerned.”* IDI-GR-02

While the majority of researchers recognized the significant value of policy briefs as a communication tool, particularly in reaching policymakers, their experiences with using policy briefs varied. Many researchers expressed poor understanding about how to develop an effective policy brief that had the likelihood of influencing policy decisions. This highlights a shared recognition among researchers of the importance of this communication approach. Additionally, they alluded to the need for training support and guidance in crafting impactful policy briefs that can effectively convey research findings to policymakers.

*“I must confess that with policy briefs, at least myself and, my team we are not using this tool as frequent as we should. And we don’t have yet the experience I mean to do that, but I do know that some of my colleagues here in the institution they are working on that.”* IDI-GR-03

*“I think that policy briefs will be better to reach them because you know because the rate of, I can say that they are more technical in some way and the language in the report must be explained in other ways. And I think that policy briefs could do this better than the actual reports because they are more tailored to inform on the potential changes that our research findings might bring, yeah”* IDI-GR-06

### Attitude towards research evidence

When asked about the reception of their research evidence in their respective countries, most researchers acknowledged that their evidence was generally well-received. However, they emphasized that there remained opportunities for improvement. Some researchers noted that the level of receptivity at the policy level was contingent upon the individuals in leadership roles. In contrast, others suggested that the perceived lack of urgency in adopting evidence from malaria research could be attributed to the endemic nature of malaria in Africa. These insights collectively underscore the nuanced factors influencing the reception and utilization of research evidence in the context of malaria control and elimination efforts.

*“I think in general there is a good receptivity because actually since in several years we have let’s say, we have a good collaboration between researchers and the policy makers but, so we try to make advocacy and we think that has improved things here.”* IDI-GR-15

*“Ok, it depends on the head of the minister (laughs). Yeah, it depends on the individual you know, you can have some who are receptive and take into account your research findings, but it depends on with whom you talked, and in which occasion you are talking and sharing your results.”* IDI-GR-10

*“I should say the major problem we face in terms of receptivity is the fact that we work in diseases that are endemic like malaria.”* IDI-GR-04

The discourse surrounding the receptivity of research evidence revealed that participants perceived an improved level of receptivity in their countries, which they attributed to the impact of the COVID-19 pandemic. They opined that COVID-19 served as a catalyst, acting as an eye-opener for policymakers, prompting them to acknowledge and utilize scientific research evidence more effectively. They articulated that the pandemic compelled policy makers and global leaders to rely heavily on researchers for evidence to inform their decision-making processes. Consequently, this unprecedented reliance on scientific research evidence during the COVID-19 pandemic has created a more conducive environment for interaction and collaboration between scientists and policymakers.

*“I mean very small fractions of the individuals would die of severe malaria or something like that, so the urgency of policy briefs was not really there, but COVID has come to teach us a lot. During the covid, the government was always looking up to our policy briefs to advice the president. So covid has come to reshape the thinking of policy makers so they are more amenable to absorbing research findings, taking them more seriously than they did pre-covid.”* IDI-GR-04

*“For me, I think it is even better now than before. With the experience with covid, it is better than before. They would engage and I think they would be open to learn, and they would be open to take up, but it must be a concerted effort everybody playing their part.”* IDI-GR-03

Participants also indicated that the quality of the evidence they produce significantly influences the responsiveness of the NMCPs. They emphasized the importance of initiating engagement opportunities between researchers and policymakers by involving the latter right from the inception of the research endeavour and maintaining collaboration throughout the process. This approach aims to enhance policymakers’ understanding of the research activities, recognizing that it is more challenging for policymakers to prioritize studies they were not involved in from the start. Establishing strong working relationships is deemed essential for the effective utilization of research evidence, and researchers noted the need to develop effective communication skills to convey their findings to policymakers. They highlighted three key related ingredients that are critical for scientist to penetrate the policy space with their study findings. First, the potential impact of the findings in question, second, the connectedness of the scientist within the policy ecosystem and third, the ability to clearly communicate the impact of the study findings.

*“The thing is, it depends on the level of evidence that you have, and it depends on the kind of relationship that you have as a scientist with the policymaker.* IDI-GR-14

*“They should also know how to communicate or how to exchange with the policy maker. This is very important. We cannot work separately, do what we want, publish, and expect change. This is very important; things are really moving, and this is now the time to really translate what we are doing in the lab into what is important to change or to adapt control strategies.”* IDI-GR-03

### Key challenges in pathogen genomic research and translation in Africa

The study explored the experiences of Malaria Genomic Researchers (MGRs) regarding the challenges encountered in conducting genomics research in Africa. The malaria genomic researchers shared insights into the challenges they encounter and how they navigate these challenges to generated evidence for policy decision-making. The detailed sub-themes provide an in-depth discussion of the various challenges faced by participants in their research endeavours, as well as the specific difficulties encountered in translating and communicating their research findings. This comprehensive exploration sheds light on the complex landscape of genomics research in the context of malaria control and elimination efforts in Africa.

Key among these challenges was access to research funding, inadequate research infrastructure, logistics (e.g. procurement), capacity and personnel instability, politics, interest in their work as well as limited to no institutional/governmental support. In what follows. These challenges are presented as sub-themes below:

### Lack of funding for research and translation

Participants expressed a general lack of funding from local organizations and government for research, particularly genomics research noting that genomics research is very expensive yet has very low interest and support from local governments in Africa. Most of the funding for genomics research across the countries is primarily from external funding sources hence, there is hardly enough funds to do the many things within the capacity of researchers and their collaborators. The cost and access to inputs for genomics research translates into other logistical and infrastructural challenges.

In particular, participants cited situations where their research activities have been adversely affected by poor access to reagents, critical equipment, and advanced machinery needed to advance their work. They expressed frustration over the fact that every little input for their research activities must be imported from the USA or Europe within a procurement context of a supply chain riddled with middle men bureaucratic resulting in massive delays in receiving procurement orders often very disruptive to their research work.

*”The work that we do really cannot be done to some extent if you don’t have sufficient funds. So, there is the obvious challenge of funds to do the work that we are doing and most of the funding comes from externally funded grants and those are not as easy to get.”* IDI-GR-11

*“And when you come to the laboratory, we do not have logistics to work, the supply chain is terrible and so we need to buy simple things and you can’t buy and the prices are high, the bureaucracy for purchasing is high. Look, we can’t finish talking about the challenges.”* IDI-GR-17

General lack of interest in research knowledge generation by stakeholders, and limited funds for research dissemination were also cited as challenges for sharing malaria genomics research findings. Participants acknowledged that malaria may not be a priority for their governments or politicians as compared to other infectious diseases. This means that scientists must be very innovative in their approaches to share their research findings to attract attention to their work. The problem however is a poor budget allocation for dissemination activities. Researchers depend on external bodies for funding and are sometimes compelled to focus on their funder’s priorities and requirements than those of their NMCPs when they have to appropriate funds.

*“I am saying that some grant money should be put aside to engage stakeholders. Sharing research findings is not cheap and requires a lot of logistics. Unfortunately, we barely have enough to complete the research and publish open access so we need funds to be able to engage properly.”* IDI-GR-13

*“If you don’t have funding, then you have to do voluntary dissemination and that is if you get the opportunity to talk either than that you may find it very difficult because they won’t come. People leaving everything they have to do and coming to your meetings would expect allowances you know to transport them to the place to listen to you*.*”* IDI-GR-11

### Inadequate research capacity

Malaria genomics researchers also highlighted a weak research support environment within which they have to operate, including limited capacity and inadequate support staff. Participants cited instances where, as scientists, they have to handle other aspects of the research process, such as managing finances, recruiting participants, handling administrative tasks, huge workload from supervising students, as well as planning and leading dissemination activities. They also highlighted a massive data processing and analysis capacity gap across the continent. They noted the large capacity gap for bioinformaticians that critical genomics data manipulation and evidence generation.

*“It’s about the capacity building because we need to have a new generation of scientist with a high quality of training and so we need to have bioinformaticians- there is a lack of bioinformaticians here in the field of malaria, so these are some challenges we have faced.”* IDI-GR-09

*“You find for example that all the students are supposed to do projects and I am the one to supervise them and I am the one reviewing all the proposals and if you ask the university to propose a supervisor to review the proposal, they end up getting corrections on the other components apart from the science. So, the critical context of the study is not touched by most of the supervisors so I can tell you so many things. Suffice to say, working in Africa is like doing five jobs and getting paid for one. (laughs)”* IDI-GR-06

### Communicating pathogen genomics to stakeholders

A consistent challenge raised in all discussions was the limited understanding of genomics by most policy makers and local communities. These researchers acknowledged that their field of research is relatively new in Africa and recognise the additional efforts required to simplify the scientific aspects of their work for better understanding and appreciation by key stakeholders. Despite their endeavours, many misconceptions around genomics persists, particularly among government officials, policy makers and research communities, as suggested in the following quotes:

*“The major challenge is just the fact that people don’t even understand why we are doing genomics. Some of the ethical reviewers raise a lot of ethical issues and all that. Aside that I think, so that is the major challenge when it comes to genomics research that people’s orientation is not high enough even though that is the direction that the science is going now.” IDI-GR-12*

*“The field of genomics is still very new, it’s still very new, there is exceptional use cases like what I just described but because the other stuff that we do are very new the NMCPs have a hard time understanding what we talk about. “IDI-GR-17*

Genomics researchers also acknowledged the inherent challenges presented by the nature of the studies they conduct. They suggested that even colleague scientists who are not genomics researchers find it difficult to understand the outputs from such research endeavours. Genomics studies are also characterised by long turnaround time for results and most of the findings are often aggregated and complex to interpret. Given the dynamic nature of the field, scientists are also cautious about sharing information because misinterpretation and/or poor communication of genomics data could have damaging effects on individuals and communities alike. Also, what may be considered relevant and accurate today may change slightly or significantly in the future.

“*Ok. So, genomics is a very dynamic field. Today this is the results we have found, and this is the implication. Tomorrow it may change slightly because you have explored different parts of the malaria parasite or the parasite itself has evolved. So, the story you told five or 6 years ago may not be the same.”* IDI-GR-07

*“But because genomics, you know, the investigations take long and the findings are aggregated so for communities, there are quite several challenges with feeding them back with some of these aggregated findings because 1, there are so many technical jargons, and it may not be that useful to them.”* IDI-GR-02

In the same vein, genomics researchers and their public engagement teams find it very difficult to translate the results of their studies into simple terms to aid understanding by stakeholders. This is particularly challenging when the audience lacks basic training in genetics and genomics. Most researchers reported encountering this challenge, especially when engaging with local communities and participants, and other stakeholders such as the NMCP and officials of the Ministry of Health. Consequently, some researchers opt not to disseminate their findings beyond publishing in peer-reviewed journals. They suggested that it is much easier to disseminate to the scientific community than all other stakeholders and attributed their struggle in translating research results effectively to a lack of capacity. They reported that most of their stakeholders especially the NMCPs in the various countries do not have the capacity to understand the magnitude of what they present nor the implications of those findings and that affects the way NMCPs receive and report malaria genomics data to higher health officials for policy making. The lack of capacity to understand makes the endeavour of translation burdensome for researchers.

*“The issue is how to let the community, or these stakeholders understand the genomics and break it down to a point where they would appreciate the complexities of the whole subject area, that is where I think the challenge is.”* IDI-GR-02

*“A lot of jargons in genomics and when you try to translate in the local language, you will have a bit of challenge in making it palatable for the local community as compared to the international community when you can easily share your findings without these challenges.” IDI-GR-04*

*“Yes, the lack of understanding by the NMCPs that is considered expert committee for the minister of health for example. If they are unable to fully grasp what it is that you are talking about, then how do they present that to the minister? If they are unable to capture or to fully grasp in this case let me say passive genetics. What you saw maybe 5 years ago is not the same today. If that concept doesn’t get understood well by the NMCP then they are also unable to package that information to the minister. And I do not sit in the minister’s meeting, so we rely on the NMCP to get findings across for us.”* IDI-GR-13

### Recommendations to improve genomics research uptake and dissemination

Participants were asked to share recommendations to improve the translation and use of genomics data for policy decision making.

Generally, genomic researchers recommended that grant holders should allocate adequate funds for research dissemination recognizing that it is an expensive venture. They acknowledged that their work is impactful on local communities only when they affect policy. They also emphasised the importance of implementing innovative approaches and strategies to feedback their research results to relevant stakeholders, to enhance understanding and attract the interest of stakeholders and broaden the public health impact of their work.

*“Ok, for recommendation, I think that we need to invest in funding human resources to do research and put more efforts and funds into translating the research for policy making. And then the government should also support research.”* IDI-GR-17

*“We are one of the few countries where the malaria control programme is completely funded domestically. So, we don’t get a lot from outside organizations. So, the researchers are either funded by the government or for example I do have some Gates funding to do some research as well but it is to answer a question that the programme wants but it was very interesting that I had a discussion with PMI and I said we need to change the funding model.”* IDI-GR-09

### Strengthening the capacity of NMCPs to engage in genomics

Researchers also recommended continuous training and education, as well as the reorientation of stakeholders in the genomics translation value chain to build and maintain interest in genomics use cases that are relevant for policy decision-making. They suggested that continuous training in various forms could enhance the knowledge of the NMCP and improve their appreciation of genomics. Consequently, this will better equip them to utilize evidence from genomics research to enhance malaria control activities.

“*I think the first will be education. Educating these stakeholders on what genomics is, and why genomics for them to understand the importance of genomics in what they do. So, for malaria control or malaria elimination, why genomics in malaria elimination? Now if they understand what it is, then the next level will be sitting down with them to come up with how they think the genomics that you’ve explained to them will be beneficial to their control or elimination strategy so that they would drive the research in genomics that we do.”* IDI-GR-01

“*I think that at the beginning of every genomic research the policy makers should always be involved. Yes, you shouldn’t wait till the research is done but you should let them know what you are going to do, the essence of what you are going to do, and the implications of your expected findings. So that they move on with you at every step of the way. So, from the start; I would have even said that at the start of the grant application.”* IDI-GR-05

Participants recommended capacity building as a crucial step in developing a critical mass of personnel within the NMCP who can effectively utilize research findings. Drawing from their experiences, they highlighted that the limited capacity to comprehend genomics significantly hampers its adoption and application. Therefore, they stressed the importance of continuous education and other training programmes aimed at enhancing the understanding of genomics among NMCP personnel and other key officials in the malaria research community who might not be well-versed in genomics and its contributions to malaria control and elimination efforts.

Researchers emphasized that engagements with NMCPs on malaria surveillance issues should focus on empowering NMCPs to engage in meaningful dialogues with researchers and funders. This approach could enable NMCPs to articulate their needs and prioritize areas for research, moving away from the conventional practice where researchers develop studies solely in response to funding calls. Additionally, capacity-building efforts for NMCPs should encompass understanding research methodologies and basic data analysis, enabling them to better grasp the scientific processes involved in research. They suggested that such a holistic approach would create a collaborative and informed environment for effective malaria control and elimination efforts.

*“Training can change the attitude of people. If you are ignorant of something you don’t appreciate that but if you persistently give this type of training to people engaged in that area, then there is a chance to listen and learn from you. So, I think an interactive type of training, like a one-day, three-day training every year may not be helpful but several within the year, planned and done around the time we are giving updates to these people in the NMCP especially with the evolving technology over time, then I think we can change the attitude of people from the NMCP.” IDI-GR-12*

*“I think that one of the best ways is to train them in terms of let’s say methodology of research in terms of how to analyze basic data because if we can open their eyes in the research area by providing some basic knowledge of research, I think that they would be more open to the discussion in malaria genomics.” IDI-GR-05*

*“Yes, this is what they want. It is not just what my funder wants to support I should be able to engage them to a point where they will say yeah, this as a science project is good but for the malaria control programme, we feel this other aspect is more of our priority than what you are talking about. So, we should be able to change strategy based on the priority of the control programme and we should also work hand-in-hand with them. We shouldn’t end up just going to have this meeting with them and we agree on the research questions, but we should walk with them through the whole process.” IDI-GR-01*

### Developing good working relationships

Another key recommendation to enhance discussions and improve the utilization of research evidence was the need to establishment and maintain good working relationships with policymakers. The researchers suggested that scientists should not adopt an “all-knowing” attitude in their interactions with National Malaria Control Programmes (NMCPs) and other stakeholders. Instead, they should demonstrate patience and humility, seeking to understand the needs and challenges of these stakeholders. By incorporating these insights into their efforts, researchers could increase the likelihood of producing data that is valuable for policy decision-making.

Additionally, they suggested that effective communication between researchers and NMCPs is crucial. As researchers learn to engage with NMCPs and the latter develops an interest in the scientific work, a shift could occur in the way they think and act in their respective roles. This increased interest could create an environment conducive to collaborative learning and sharing, fostering the development of policies based on mutual understanding. For instance, merely inviting NMCPs to a workshop may not be as impactful as building genuine interest and encouraging them to actively participate in a training workshop. The researchers noted that this approach emphasizes the importance of cultivating a shared interest and commitment for meaningful collaboration in policy development.

*“We should work together. We should really work together. Each of us has to know what the main steps and the processes of each path at the policy maker level are and at the researcher level also.” IDI-GR-13*

*“I think that we have to invite them during our genomic scientific activities let’s say meeting and try to discuss on the need and also to share with them our objective and get the feedback to know how we can implement the different activities in the country.” IDI-GR-05*

### Emulating best practices and learning from others

Another key recommendation to enhance discussions and improve the utilization of research evidence was the need to establishment and maintain good working relationships with Demonstrating examples of how genomics data has been utilized to enhance malaria interventions in different nations can be beneficial, particularly when seeking the support of NMCPs. In instances where internal bureaucratic processes pose challenges, researchers should consider collaborating with external organizations like the World Health Organization (WHO) or the Africa Centres for Disease Control and Prevention (Africa CDC). Sharing research findings with such external entities could result in policy recommendations being conveyed back to countries in Africa, which are more likely to be heeded and put into practice.

*“One thing that I think would work or I have seen work best is to get cases, I mean to show that another country did something in a particular way that had an impact I mean, they will get caught up if they need to learn how to do next generation sequencing and analysis,” IDI-GR-16*

*“Sometimes it takes, it takes the involvement of WHO, because the NMCPs, they usually, they like to follow whatever WHO tells them. So sometimes you have to go and convince WHO, and then WHO makes a recommendation that comes back to the country and then it becomes adopted. But there have been cases where we have been able to influence policy that has not gone through WHO.” IDI-GR-03*

## Discussion

According to participants of the current study, NMCPs do not fully understand the implications of the evidence from their genomics data. This has resulted in very minimal use of genomics data for malaria control and elimination in most of the countries. This finding is consistent with a study in the Greater Mekong subregion which also found that NMCPs do not adequately use parasite genetics data to inform their programme activities and planning [12]. It is observed that NMCPs’ lack of understanding of genomics translates into devaluation or a lack of appreciation of rich evidence produced through the research activities of the malaria scientists. Consistent with a study in Malawi, participants of the current study reported that policy makers are likely to underappreciate research evidence where they have no interest or understanding of such. They suggest that policy makers should be part of the entire process of data generation to ensure they understand the processes and bottlenecks that researchers navigate to produce the evidence they share [14]. Accordingly, once policy makers understand all the processes researchers go through and the amount of work they put in to produce quality evidence, their attitude to research evidence uptake and use may improve including research findings they may not have immediate use for.

Varied levels of interaction are seen across the countries. While some researchers are enjoying valuable working relations with the malaria policy makers and NMCPs in their countries, other researchers are yet to have the full support of their institutions. This varied level of interest in the work of the researchers also makes it very difficult for researchers to benefit fully from international collaborations. The lack of interest could be driven by several factors including politics, other government priorities and lack of commitment to the fight against malaria considering there are other infectious diseases in Africa that need attention than endemic malaria [15, 16]. While science is evolving and most people including malaria policy makers and the NMCPs may have diverse ideas of genomics and its value to malaria control and elimination, the current study suggests a weak link between producers and users of research evidence consistent with a study in Southeast Nigeria on the barriers and solutions for using research evidence for endemic disease elimination [16].

The study suggests that employing innovative methods of engaging policy makers and the NMCP through meaningful engagement and transparency could enhance their receptivity of researchers’ outputs. Nonetheless, participants of the current study acknowledge certain challenges associated with collaborating with non-technical individuals in research such as time constraints, extended decision-making period, and discouraging attitudes. Despite these challenges, they emphasized that persisting with these collaborative engagements could improve uptake of evidence and promote a “learning by doing” approach to efficient data sharing for policy improvement [17].

The frequency of communication between researchers and the NMCP reflects the level of interaction they have. While the use of media is applauded for the sharing of information, there are constraints and calls to be cautious with what information is disseminated. Policy briefs have however been cited as the most effective tool for translating research findings to policy makers in some studies and this is consistent with the findings of this current study. The value of the policy brief is in the ability of the researcher to succinctly state the policy implication of the key finding and suggest recommendations [17, 18].

There is no one right way to handle the interaction between genomics researchers and NMCPs. Researchers have resorted to using external partner bodies such as the WHO and the Africa CDC to push for the uptake of their research evidence where their local country policy makers are reluctant to listen to them. Most participants of the current study are not happy with the fact that in-country policy makers do not trust their findings but are happy to accept and implement policy recommendations from the same findings when they are brought back to the country through the external partners [14, 17]. There is a potential opportunity of engaging with policy makers to understand their fears and reservations with the evidence generated in-country to properly address them and promote collaboration.

The challenges of conducting malaria genomics research are enormous and widely researched. While researchers struggle with issues of logistics and funding to conduct their research, they are also faced with problems of lack of interest and poor understanding of the work they do in their effort to translate or disseminate their findings. This is particularly challenging and a source of discouragement to researchers because the very people who could take and use their research are those with low interest in their work because of their inability to understand the work they do [14, 18]. Studies have proven that policy makers are likely to appreciate and use research evidence if such evidence is properly articulated and presented [17] and their capacities built to know and experience the basics of whatever evidence is presented to them [18]. While it may be an unfruitful venture to attempt training NMCP personnel as geneticists, it would be worthwhile to enhance their capacity in basic genetics [14, 19]. Improved capacity breeds interest and understanding which are catalysts for utilization as shown in this current study and consistent with a study of research and evidence-based decision-making in Nigeria [18].

While most of the recommendations shared by participants of the current study are feasible, their successful implementation will depend on the willingness and attitudes of both researchers and policymakers to establish and maintain shared foundations for generating and utilizing evidence in the context of malaria control and elimination. Enhancing communication strategies, such as creating policy briefs, aligns with findings from previous studies focused on informing policy decisions through research [17, 20]. Continuous training and education as well as re-orientation of stakeholders to build interest for co-sharing and co-learning as part of the recommendations to promote effective interaction between malaria genomics researchers and malaria policy makers may be unique to the current study, but there are similar calls for such collaborative discourse in Neglected Tropical Diseases in Africa [18].

### Implications of findings for policy and future research

The findings of the current study carry significant policy implications:

First, the challenges identified in the conduct and translation of malaria genomics research in Africa offer valuable insights for the governments of PDNA member countries. This information can be used to guide policies aimed at enhancing the research landscape within their nations. Addressing these challenges could contribute to more effective and impactful malaria genomics research, ultimately supporting broader efforts in malaria control and elimination. Secondly, the recommendations regarding capacity building for NMCPs provide actionable guidance for governments and funding agencies. Recognizing the importance of capacity building in leveraging research evidence, governments and funders may consider allocating resources to strengthen the capabilities of NMCPs. This targeted funding can enhance the utilization of genomics data for informed policy decision-making, aligning with control and eliminate malaria in Africa. These findings offer practical insights that can inform policy decisions, fostering improvements in research practices and capacity-building efforts to advance malaria control and elimination initiatives in the African context.

Based on these findings, the study makes recommendations for future research to quantitatively measure the level of engagement between National Malaria Control Programmes (NMCPs) and researchers in Pathogen Genomic Diversity Network Africa (PDNA) member countries. This measurement could provide valuable data to inform targeted efforts in bridging the gap between researchers and policymakers. Additionally, the need to explore the impact of these experiences and challenges on malaria control and elimination in Africa should form part of future research plans and endeavours. This entails delving deeper into how the identified challenges and experiences of Malaria Genomic Researchers (MGRs) may influence broader efforts in the fight against malaria. Investigating the experiences of NMCPs to gain a comprehensive understanding of their challenges in utilizing genomics data for policy decision-making needs to be prioritized. This holistic approach would contribute to a more nuanced and well-rounded perspective, ensuring that future studies capture the perspectives of both researchers and policymakers in the realm of malaria genomics research and its translation into policy and practice.

## Strengths and limitations

A major strength of this study is the opportunity it provides in consolidating insights from various African countries in a single research endeavor. The main limitation of this study is the absence of the perspectives and experiences from National Malaria Control Programmes (NMCPs). Although the study offers valuable perspectives from Malaria Genomic Researchers (MGRs), the absence of corresponding insights from NMCPs hinders a comprehensive understanding of the addressed issues. Incorporating NMCPs’ experiences could provide a more comprehensive and nuanced perspective, and a holistic view of the challenges and opportunities in translating genomics research into policy and practice. Nonetheless, the findings from the study have highlighted important gaps in the literature and provided some key points to consider accelerate the translation of genomics data into policies and programmes to support malaria elimination in Africa.

## Conclusion

This study has highlighted the experiences of malaria genomics researchers and the challenges they face in translating research for use by policy makers. It has also revealed the limited interest and capacity on the part of policy makers to understand and use genomics data and suggest innovative engagement approaches to build good relationships that promote co-sharing and co-learning between researchers and policy makers. The findings underscore the critical importance of allocating funds specifically for well-planned dissemination activities to ensure the impactful translation of research. It is emphasized that these dissemination activities should be organized effectively, coupled with incentives, to capture and maintain the interest of NMCPs. The study further highlights the necessity for scientists to carefully and innovatively develop communication strategies, recognizing the evolving nature of science. It emphasizes the importance of not assuming a lack of capacity among stakeholders to understand genomics, as there is an increasing appreciation for this field. It also recognises that researchers acknowledge the inherent challenges in conducting malaria genomics research in Africa and are willing to invest in capacity building. The focus is on promoting understanding, utilization, and effective translation of research evidence into actionable messages as well as continuous education on the efficacy of Malaria Molecular Surveillance (MMS) as a tool in the fight against malaria. Additionally, it advocates for fostering collaborative efforts between malaria researchers and policymakers across Pathogen Genomic Diversity Network Africa (PDNA) countries and sub-Saharan Africa at large.

## Data Availability

No datasets were generated or analysed during the current study.

## Abbreviations

PDNA: Plasmodium Diversity Network, Africa
NMCP: National Malaria Control Programme
WHO: World Health Organization
MGR: Malaria Genomic Researchers
MMS: Malaria Molecular Surveillance
SMC: Seasonal Malaria Chemoprevention
IRS: Indoor residual spraying
IPTP: Intermittent preventive treatment in pregnancy

## References

[1] WHO. Compendium of WHO malaria guidance: prevention, diagnosis, treatment, surveillance and elimination. Geneva: World Health Organization; 2019.

[2] WHO. World malaria report 2023. Geneva: World Health Organization; 2023.

[3] Wagman J, Cissé I, Kone D, Fomba S, Eckert E, Mihigo J, et al. Rapid reduction of malaria transmission following the introduction of indoor residual spraying in previously unsprayed districts: an observational analysis of Mopti Region, Mali, in 2017. Malar J. 2020;19:340. 32950056 10.1186/s12936-020-03414-2 PMC7501620

[4] Apinjoh TO, Ntui VN, Chi HF, Moyeh MN, Toussi CT, Mayaba JM, et al. Intermittent preventive treatment with Sulphadoxine-Pyrimethamine (IPTp-SP) is associated with protection against sub-microscopic *P. falciparum* infection in pregnant women during the low transmission dry season in southwestern Cameroon: a semi-longitudinal study. PLoS ONE. 2022;17: e0275370. 36178962 10.1371/journal.pone.0275370 PMC9524640

[5] Chatio S, Ansah NA, Awuni DA, Oduro A, Ansah PO. Community acceptability of Seasonal Malaria Chemoprevention of morbidity and mortality in young children: a qualitative study in the Upper West Region of Ghana. PLoS ONE. 2019;14: e0216486. 31100072 10.1371/journal.pone.0216486 PMC6524792

[6] Coulibaly A, Diop MF, Kone A, Dara A, Ouattara A, Mulder N, et al. Genome-wide SNP analysis of *Plasmodium falciparum* shows differentiation at drug-resistance-associated loci among malaria transmission settings in southern Mali. Front Genet. 2022;13:943445. 36267403 10.3389/fgene.2022.943445 PMC9576839

[7] Salomão C, Sacarlal J, Gudo ES. Assessment of coverage of preventive treatment and insecticide-treated mosquito nets in pregnant women attending antenatal care services in 11 districts in Mozambique in 2011: the critical role of supply chain. Malar J. 2017;16:223. 28545540 10.1186/s12936-017-1872-2 PMC5445451

[8] van Eijk AM, Hill J, Alegana VA, Kirui V, Gething PW, ter Kuile FO, et al. Coverage of malaria protection in pregnant women in sub-Saharan Africa: a synthesis and analysis of national survey data. Lancet Infect Dis. 2011;11:190–207. 21273130 10.1016/S1473-3099(10)70295-4 PMC3119932

[9] Hemingway J, Shretta R, Wells TNC, Bell D, Djimdé AA, Achee N, et al. Tools and strategies for malaria control and elimination: what do we need to achieve a grand convergence in malaria? PLoS Biol. 2016;14: e1002380. 26934361 10.1371/journal.pbio.1002380 PMC4774904

[10] Crawley J, Hill J, Yartey J, Robalo M, Serufilira A, Ba-Nguz A, et al. From evidence to action? Challenges to policy change and programme delivery for malaria in pregnancy. Lancet Infect Dis. 2007;7:145–55. 17251085 10.1016/S1473-3099(07)70026-9

[11] Mwenesi H, Mbogo C, Casamitjana N, Castro MC, Itoe MA, Okonofua F, et al. Rethinking human resources and capacity building needs for malaria control and elimination in Africa. PLoS Glob Pub Health. 2022;2: e0000210. 36962174 10.1371/journal.pgph.0000210 PMC10021507

[12] Jacob CG, Thuy-Nhien N, Mayxay M, Maude RJ, Quang HH, Hongvanthong B, et al. Genetic surveillance in the Greater Mekong subregion and South Asia to support malaria control and elimination. Elife. 2021;10: e62997. 34372970 10.7554/eLife.62997 PMC8354633

[13] Lumivero. NVivo (Version 12). 2023. www.lumivero.com. Accessed 19 July 2023.

[14] Mwendera CA, de Jager C, Longwe H, Phiri K, Hongoro C, Mutero CM. Facilitating factors and barriers to malaria research utilization for policy development in Malawi. Malar J. 2016;15:512. 27760552 10.1186/s12936-016-1547-4 PMC5070004

[15] Guyant P, Corbel V, Guérin PJ, Lautissier A, Nosten F, Boyer S, et al. Past and new challenges for malaria control and elimination: the role of operational research for innovation in designing interventions. Malar J. 2015;14:279. 26185098 10.1186/s12936-015-0802-4 PMC4504133

[16] Ezenwaka U, Mbachu C, Etiaba E, Uzochukwu B, Onwujekwe O. Integrating evidence from research into decision-making for controlling endemic tropical diseases in South East Nigeria: perceptions of producers and users of evidence on barriers and solutions. Health Res Policy Syst. 2020;18:4. 31931821 10.1186/s12961-019-0518-y PMC6958705

[17] Damba FU, Mtshali NG, Chimbari MJ. Barriers and facilitators of translating health research findings into policy in sub-Saharan Africa: a scoping review. Humanit Soc Sci Commun. 2022;9:65.

[18] Ezenwaka U, Onwujekwe O. Getting evidence from health policy and systems research into policy and practice for controlling endemic tropical diseases in Nigeria: assessing knowledge, capacity, and use. Front Trop Dis. 2021;2:735990.

[19] Noviyanti R, Miotto O, Barry A, Marfurt J, Siegel S, Thuy-Nhien N, et al. Implementing parasite genotyping into national surveillance frameworks: feedback from control programmes and researchers in the Asia-Pacific region. Malar J. 2020;19:271. 32718342 10.1186/s12936-020-03330-5 PMC7385952

[20] Mbonye AK, Magnussen P. Translating health research evidence into policy and practice in Uganda. Malar J. 2013;12:274. 23915001 10.1186/1475-2875-12-274 PMC3750267

